# Cases Pathologically Illustrative of the Distinct and Separate Nervous Functions Subservient to Voluntary Motion and Feeling

**Published:** 1827-05

**Authors:** S. D. Broughton

**Affiliations:** Surgeon to His Majesty's 2d Regiment of Life Guards, and to the St. George's and St. James's Dispensary.


### FUNCTIONS OF THE NERVES.

*Cases pathologically illustrative of the distinct and separate Nervous Functions subservient to Voluntary Motion and Feeling.*

By S. D. Broughton, Surgeon to His Majesty's 2d Regiment of Life Guards, and to the St. George's and St. James's Dispensary.

Case I.—E. Bundy, a dragoon farrier, about twenty-seven years of age, was admitted into the regimental hospital, November 27th, 1826, complaining of slight pain about the forehead and temples, and throughout the limbs. His tongue was pale and moist; the pulse about ninety, small and soft; the eyes had a natural appearance.

℞. Haust. Sennæ comp. stat. sumend.—℞. Pulv. Ipecac. comp., Pulv. Jacobi, āā ℈ss. M. capiat nocte.

Nov. 28th.—The bowels have been freely open; the pulse is reduced in frequency, and he is relieved from the pains.

Rep[r] Haustus.—Rep[r] Pulv. ter die sumend.

Nov. 29th.—This morning, on waking, the pain across the forehead returned, and there was a disposition to drowsiness. His speech is indistinct and somewhat incoherent, and his pulse is increased in frequency. His tongue is moist and whitish, and there is some moisture on the skin generally. He has evidently a slight difficulty in adjusting the muscles of the face and mouth. The pupils contract and dilate. *He has the perfect use of all his limbs;* but, while the muscles are obedient to the will, *the integuments of the left side of the body, from the top of the head to the extremity of the toes, are absolutely insensible to the application of a pin, with which the skin has been punctured in several places, besides inserting it between the nail and flesh of the great toe.*

After shaving the crown of the head, a large blister was applied, and twenty leeches placed on the temples. The following draught was given every four hours—

℞. Misturæ Salinæ ℥jss.; Vini Antim. Tart. ʒj.; Magn. Sulph. ʒss. M.

He took these pills at night—

℞. Hydrarg. Submuriat., Pil. Aloetic. comp. āā gr. v. M. fiant pil. ij.—The powders are omitted.

30th.—The drowsiness has gone off, and his speech is improved: the pulse is at eighty-four; the bowels are loose. The pain of the head is removed, and there is no incoherence. The tongue is more natural in appearance. The skin is insensible to feeling as yesterday.

December 1st.—A great improvement in his speech is evident this morning. The pulse is at seventy-two; the bowels are loose. The sensibility of the integuments seems to be partially restored. He sat up in the evening, and could walk perfectly well, though some numbness remained.

Dec. 14th, (eighteen days after his admission,) he was dismissed the hospital, having quickly regained his freedom of speech, and gradually the sensibility of the skin; which, however, was not perfectly restored at the period of his discharge from the hospital. But, on his return from leave of absence, during which he was enjoined to live low and quietly with his family, the sense of feeling seemed to be equal on both sides of the body, and he is doing his duty as usual.

Case II.—J. Church, a trooper, formerly addicted to drinking, was admitted into the regimental hospital, March 12th, 1825. The day previous, he was attacked with sudden pain at the back of the head and across the forehead, inclining to the right side. *The forearm and foot of the same side were chiefly affected, and rendered entirely incapable of voluntary motion; but the skin was perfectly sensible all over the body.* Giddiness attended the commencement of the attack, and a sense of coldness was referred to the motionless limbs. The bowels are open, and the tongue is furred. There is a slight distortion of the muscles of the face on the right side, and the speech is slightly impeded. The pupils contract and dilate; the pulse is slow, soft, and equable; the evacuations are dark green.

℞. Hydrarg. Submuriat. gr. v.; Pil. Colocynth. comp. ℈ss. M. fiant pil. iij. nocte sumend.—℞. Aquæ Cinnamomi ℥jss.; Magnesiæ Sulph. ʒij.; Træ. Cinnam. comp. ʒjss. M. capiat sextis horis.—Subsequently five grains of the Bluepill were given every night, with the above draught by day.

March 21st.—He has continued to get gradually better, and felt immediate relief from purging. Some degree of motion is restored to the forearm and foot, and the sensibility of the skin has continued unimpaired.

A stimulating liniment is used, and the gentle use of the pulley is daily adopted.

Shortly afterwards he was made a Chelsea pensioner; but his limbs had only partly regained their motion.

Case III.—G. Rhodes, a trooper, about thirty years of age, was admitted into the regimental hospital, February 13th, 1824. He was seized, while on guard, with giddiness and pain of the head, the night before his admission. His tongue is furred, and the bowels costive. *He can use his limbs, but the integuments of the left side are insensible.* His pulse is at ninety, and rather full.

About twenty ounces of blood were taken from the arm; and a brisk purge was administered; ten grains of Calomel and ten grains of James's Powder were given at night.

Feb. 14th.—Some pain of the head remains, but he is better upon the whole.

Fifteen leeches were applied to the temples.—℞. Misturæ Salinæ ℥jss.; Vini Antim. Tart. ʒj. M. capiat quartis horis.

15th.—The insensibility of the skin continues, with a sense of coldness.

℞. Hydrarg. Submuriat. cum Scam. ℈ss.; Pulv. Antimon. gr. v. M. fiant pil. iij. nocte maneque sumend.

18th.—Has been improving, but complains to-day of sickness and oppression at the epigastrium, which was relieved by an emetic; and his tongue, previously foul, became clean. The mixture is omitted. His evacuations became more natural under the use of the above and of the following pills. He has been free from any appearance of fever.

Instead of the last pills, the following were prescribed—℞. Pil. Hydrarg. gr. v.; Pulv. Rhei gr. v. M. fiant pil. iij. nocte sum. And the following draught was given three times a-day—℞. Infus. Gent. comp. ℥ jss.; Magnesiæ Sulph. ʒ jss. M.

The sensibility of the skin returned with the improvement of his general health.

Case IV.—G. Monkton, about twenty-eight years of age, a trooper, was admitted into the regimental hospital, December 2d, 1823. While on guard, he had been seized with loss of motion on the right side of the body. There was a slight numbness and cold sensation in the face, with a little difficulty in adjusting the fascial muscles; but the puncture of a pin was felt throughout the body. The tongue was white; the pulse small and quick; the bowels were open; the face and eyes had a yellow appearance; and the feces were not coloured. He had been standing as sentry during a remarkably severe frost.

He was immediately put into a hot bath, and took the following pills—℞. Pil. Colocynth. comp ℈ss.; Hydrarg. Submuriat. gr. v. M. fiant pil. iij.

Dec. 3.—Relieved, but the insensibility of the skin remains.

4th.—A dull pain exists at the occiput, and a disposition to giddiness on moving up the head. Bowels open, and the motions improved in colour. The tongue is less furred, and the pulse quiet.

The hot bath is repeated, and a few leeches previously applied to the temples.—Rep^r Pilulæ.

5th.—A sense of coldness has been complained of in the face, and the skin remains insensible. Bowels open, and stools less pale.

℞. Inf. Calumbæ ℥ jss.; Træ. Rhei ʒ ij.; Træ. Cinnam. comp. ʒ j. M. capiat ter die.—℞. Pil. Hydrargyri gr. x. noctibus sumend.

6th, 7th, 8th, 9th, 10th, and 11th.—He has decidedly gained ground the last few days: the yellowness of the face and eyes is going off, and his evacuations assume a proper colour and odour. Appetite improved; pulse quick; no pain or giddiness of the head; and the sensibility of the skin is returning. He can now adjust the muscles of the face and mouth; but a sense of numbness and coldness still remains.—Pergat.

15th.—He has been going on very well till to-day, when a degree of double vision was noticed, and the right iris was contracted. Bowels open.—Pergat.

17th.—The contraction of the iris is removed. He complains of dizziness. Bowels moderately open.

Twelve leeches were yesterday applied to the temples.

19th.—Dizziness continues, and uneasiness at the epigastrium.

An emetic was given, and he was relieved.—Pergat.

22d.—He has been going on well, and is always better when the bowels are loose. The sense of coldness, and insensibility of the right side of the face, are removed.

A strong liniment has been daily used; and the Pil. Hydrarg. sometimes given two or three times a-day, with the bitter aperient draughts.

On the 1st of March he went on leave of absence, and shortly after was allowed to purchase his discharge; and continues in good health, but was for some time disposed to be bilious.

Case V.—A gentleman in the cavalry, aged twenty-four, while in the South of France, during the autumn of 1825, fell sixteen feet into the dry bed of a river, and pitched first on the right hip. He found himself perfectly sensible and collected, and free from pain, but totally incapable of moving his lower limbs, or of standing when placed upright. He was conveyed home, and remained some time confined to his bed. During this period, *the skin over the nates and part of the loins, the perineum, the penis, and the upper part of the thighs, was rendered entirely insensible to feeling, so that a pin might be inserted without the least degree of sensation;* and, while this insensibility was coming on, he regained the power of moving his lower limbs by slow degrees, the skin of these never having lost their feeling. When after some time he was able to get out of bed, the flesh of the thighs, legs, and feet had wasted, and the limbs become smaller, with very limited powers of motion, by short dragging steps, and assisted by a stick. At first there was great inflammation of the glands in the groin, and about the urethra, with much pain extending along the perineum. Subsequently, these parts becoming insensible to feeling, he was scarcely conscious of the presence of the catheter. The urine, for some time after the accident, emitted a disagreeable odour, and was constantly thick and muddy. The feces came away unconsciously to the patient.

In March following, he returned to England. His gait was perceptibly awkward, and impeded by short and unfirm steps. The feet, legs, and thighs were less than usual in size; the skin over them was perfectly sensible; but, from below the small of the back to the top of the thighs, the skin, including that of the penis, was quite insensible to feeling. Erections of the penis came on frequently during sleep, and often accompanied by emission without sensation. When the bladder was full, the urine overflowed on the slightest irregular movement, and run out of the penis unconsciously to the patient. The feces continued in the large intestines, unless they were liquid, and he had no voluntary power to assist their expulsion, being obliged to take medicine to promote their passage.

In the course of last year, the limbs assumed a fuller condition, the step became bolder and more measured, and he could manage a horse. His powers of urinary retention and expulsion gradually returned, and the feces were properly passed; but the *insensibility of the integuments remains with little improvement to this day, a sense of numbness continuing in the skin of the perineum and nates;* while his locomotive powers, and development of flesh in the lower limbs, are as good as ever. The Ung. Antimon. Tart., electricity, and strong stimulating liniments, have been applied to the loins without any benefit; but his general health seems to have been improved by the shower-bath.

---

